# Safety and efficacy of convalescent plasma therapy in severely and critically ill patients with COVID-19: a systematic review with meta-analysis

**DOI:** 10.18632/aging.202195

**Published:** 2020-12-15

**Authors:** Luo Wenjing, Feng Yuanzheng, Jun-Ying Li, Liang V. Tang, Hu Yu

**Affiliations:** 1 Institute of Hematology, Union Hospital, Tongji Medical College, Huazhong University of Science and Technology, Wuhan 430022, China

**Keywords:** COVID-19, SARS-CoV-2, convalescent plasma therapy, systematic review, meta-analysis

## Abstract

Background: The rapidly evolving coronavirus disease 2019 (COVID-19) has resulted in more than 24 million infections and 821 thousand deaths. However, a vaccine or specific drug is absent up to this date and more attention has been focused on the use of convalescent plasma (CP). Several articles have described the CP treatment for patients with SARS-CoV-2 infection. But a comprehensive systematic review with meta-analysis about the safety and efficacy of CP transfusion in SARS-CoV-2-infected patients has not been published. We conducted this study for a better understanding of the therapeutic significance of CP for patients with COVID-19.

Results: A fixed-effect model (I^2^=0.0%) was used on the 9 articles for quantitative analysis showing that the mortality of patients with COVID-19 treated with or without CP was statistically significant (RR=0.57 [0.44-0.74]). Subgroup analysis showed that the severely ill patients benefited more from CP than the critically ill patients. Our study concluded that clinical improvement in severe COVID-19 cases were obvious. Adverse events were few and the effect of convalescent plasma on reducing viral load was apparent.

Conclusions: Convalescent plasma therapy appears safe for COVID-19, and plasma treated patients have marked reductions in their serum viral loads and most are virus negative after transfusion. Patients with severe COVID-19 benefit more from the convalescent plasma transfusion than critical patients, and patients treated in early stage are more likely to survive.

Methods: We reviewed the scientific literature from four databases published from December 8, 2019 to August 20, 2020. Statistical analyses were performed with STATA (version 15.1; Stata Corporation, College Station, TX, USA). The frequency with 95% confidence intervals (CI) was assessed using fixed effect model in analyzing the overall mortality and p <0.05 was considered statistically significant.

## INTRODUCTION

The outbreak of severe acute respiratory syndrome 2, which was caused by a novel coronavirus, SARS-CoV-2, has high rates of transmission and its mortality is about 2.3% for all cases, 25.5% for severe cases [1] and 49.0% for critical cases [2], and has thus posed significant threats to global health and economy [3]. As of 28^th^ August 2020, according to the World Health Organization Situation Report, this epidemic had spread to more than 216 countries with 24 021 218 confirmed cases, including 821 462 deaths [4]. It is a pity that no vaccine or specific drug to this highly contagious disease has been developed to date. As we all know, the SARS-CoV-2 virus belongs to the family of coronaviruses, which also comprises the Middle East Respiratory Syndrome virus (MERS-CoV) and SARS-CoV that caused previous respiratory syndrome outbreaks. However, this is the first pandemic caused by a member of the coronavirus family [5]. Based on the experience of treating these viral infectious disease, it might be worthwhile to test the safety and efficacy of convalescent plasma transfusion in SARS-CoV-2-infected patients [6]. Laboratory-confirmed patients who had fully recovered and been discharged from the hospital for more than 2 weeks, were recruited, from whom plasma was collected to treat currently-infected COVID-19 patients. The collected plasma was laboratory-examined and the procedure was supervised by clinicians [7].

Now different types of articles, including case reports, case series, observational studies, randomized clinical trials and others, have addressed the safety and the effectiveness of convalescent plasma therapy in patients with COVID-19. We selected the literature meeting the inclusion criteria from four databases to explore the significance of convalescent plasma therapy systematically and give some advice for clinical treatment.

## RESULTS

27 publications [8–34] met our inclusion criteria, including case reports, case series, observational studies, randomized clinical trials and others as described previously. The detailed information of the 9 studies [8, 18, 20, 21, 24–27, 34], we analyzed to study the mortality in patients with COVID-19, is shown in Table 1. Based on the assessment of quality, the included studies had a low risk of bias (Table 2a and 2b).

**Table 1:** The characteristic of the 9 articles included for analyzing mortality of COVID-19.

**Author**	**Journal**	Date***	**City**	**Time Enrollment**	**Disease Severity**	**CP group**		**Control group**	
						**Death**	**Total**	**Death**	**Total**
Michele L Donato	medRxiv	2020.8.4	NYC	2020.4.15-2020.6.18	severely ill	7	15	217	317
Arvind Gharbharan	medRxiv	2020.7.3	Netherlands	2020.4.8-?	NULL	6	43	11	43
Anwar M. Rasheed	medRxiv	2020.6.30	Baghdad. Iraq	2020.4.3-2020.6.1	critically ill	1	21	8	28
Hassan Abolghasemi	Transfusion and Apheresis Science	2020.6.25	Iran	2020.3-2020.4	moderate-severe	17	115	18	74
Xia xinyi	Blood	2020.8.6	Wuhan, China	2020.2.4-2020.3.20	severely ill & critically ill	3	138	59	1430
Cesare Perotti****	haematologica	2020.7.23	Italy	2020.3.25-2020.4.21	severely ill	3	46	7	30
Li Ling	JAMA	2020.6.3	Wuhan, China	2020.2.14-2020.4.1	total	8	51	12	50
					severely ill	0	23	2	22
					critically ill	8	28	10	28
Zeng Qinglei	The Journal of Infectious Diseases	2020.3.28	Zhengzhou, China	NULL	critically ill	5	6	14	15
Sean T. H. Liu*	medRxiv	2020.3.22	NYC	2020.3.24-2020.4.8	severely ill	5	39	38	156
Sean T. H. Liu**	medRxiv	2020.3.22	NYC	2020.3.24-2020.4.8	severely ill	5	39	17	78

*The article by Sean T. H. Liu et al was a matched control study, and we extracted the 2 different matched groups of mortality data. And we calculated the death of control group through the mortality described in this paper. This represents that the included patients were 1:4 (CP : control) matched. **This represents that the included patients were 1:2 (CP : control) matched. ***Date represents the time when the article was received or published. ****The primary outcome of this study was 7-days hospital mortality, and we used this data roughly. We analyzed the mortality after excluding this study, and the result showed us that the reduced mortality remained statistically significant.

**Table 2a:** Quality assessment of included studies by Cochrane collaboration’s tool for assessing risk of bias.

**Author**	**A**	**B**	**C**	**D**	**E**	**F**	**G**
Li Ling	low risk	low risk	high risk	low risk	low risk	low risk	low risk
Arvind Gharbhara	low risk	unclear	high risk	low risk	low risk	low risk	low risk

A: Sequence generation; B: Allocation concealment; C: Blinding of participants, personal; D: Blinding of outcome assessors; E: Incomplete outcome data; F: No selective outcome reporting; G: Other sources of bias.

**Table 2b:** Quality assessment of included studies by the Newcastle-Ottawa Scale (maximum score of 9).

**Author**	**Selection**			**Comparability**			**Outcome**		**ALL**
	**Represent-ativeness of the Exposed Cohort**	**Selection of the Exposed Cohort**	**Ascerta-inment of Exposure**	**Demon-stration That Outcome of Interest Was Not present at start of Study**	**Comparability of cohorts on the basis of the design or analysis**	**Assess-ment of Outcome**	**Was Follow-Up Long Enough for Outcome to Occur**	**Adequacy of Follow Up of Cohorts**	
Anwar M. Rasheed	*	*	*	*	**	*	*	*	9
Michele L Donato	*	*	*	*	*	*	*	*	8
Sean T. H. Li	*	*	*	*	**	*	*	*	9
Cesare Perott	*	*	*	*	**	*	*	*	9
Qinglei Zeng	*	*	*	*	**	*	*	*	9
Hassan Abolghasem	*	*	*	*	**	*	*	*	9
Xinyi Xia	*	*	*	*	**	*	*	*	9

### The effect of CP on mortality in patients with COVID-19

**Figure 1:**
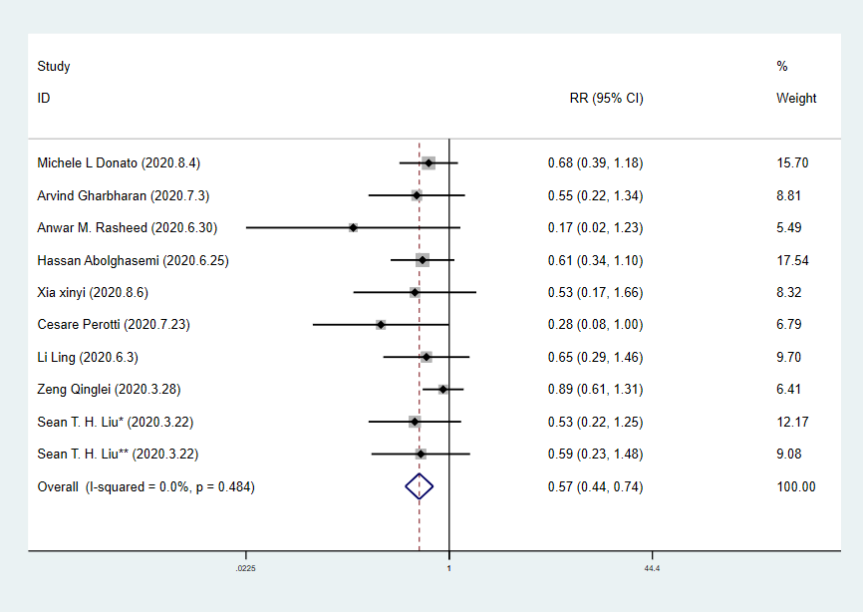
**Forest plot of RR for mortality in patients with COVID-19.**

**Figure 2:**
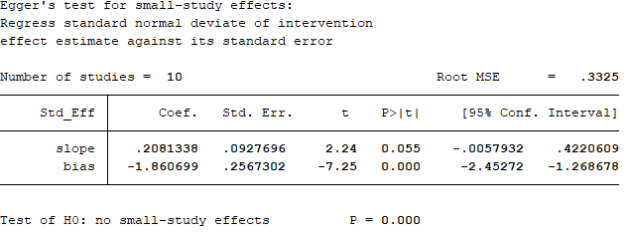
**Egger’s test for the publication bias.**

**Figure 3:**
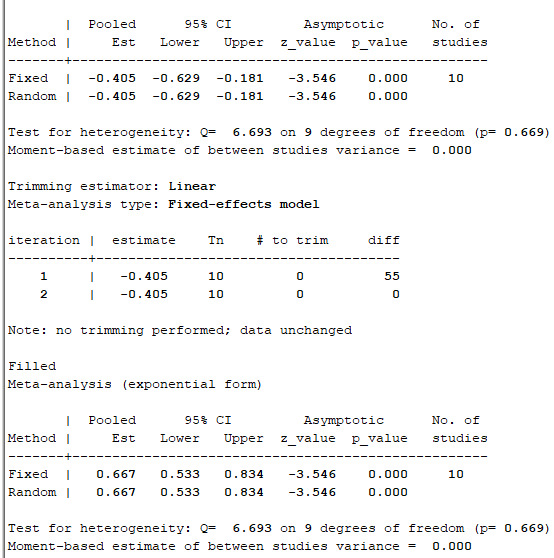
**Trim and fill method with fixed-effects model to further examine the publication bias.**

**Figure 4:**
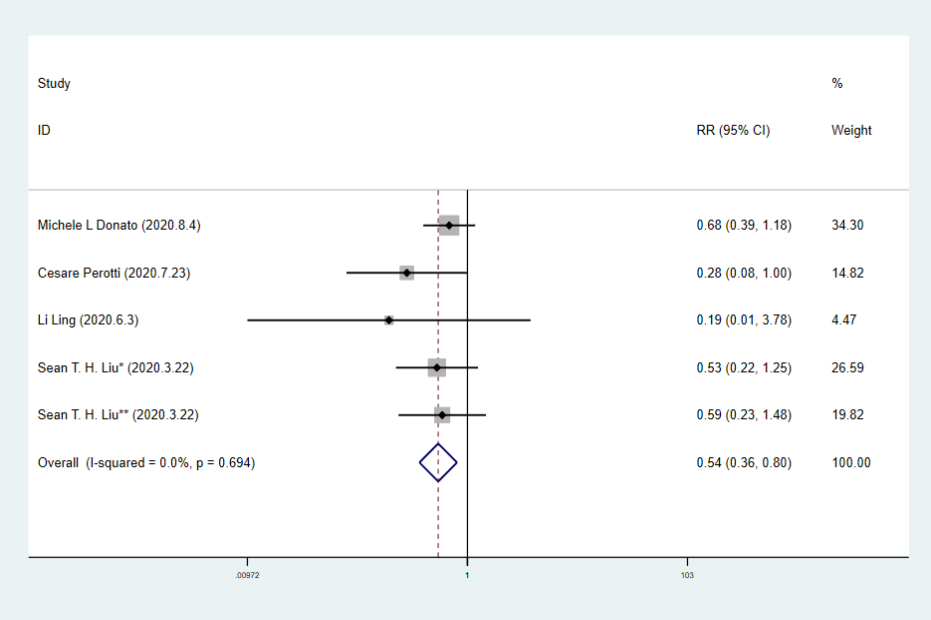
**Forest plot of RR for mortality in severe patients with COVID-19.**

**Figure 5:**
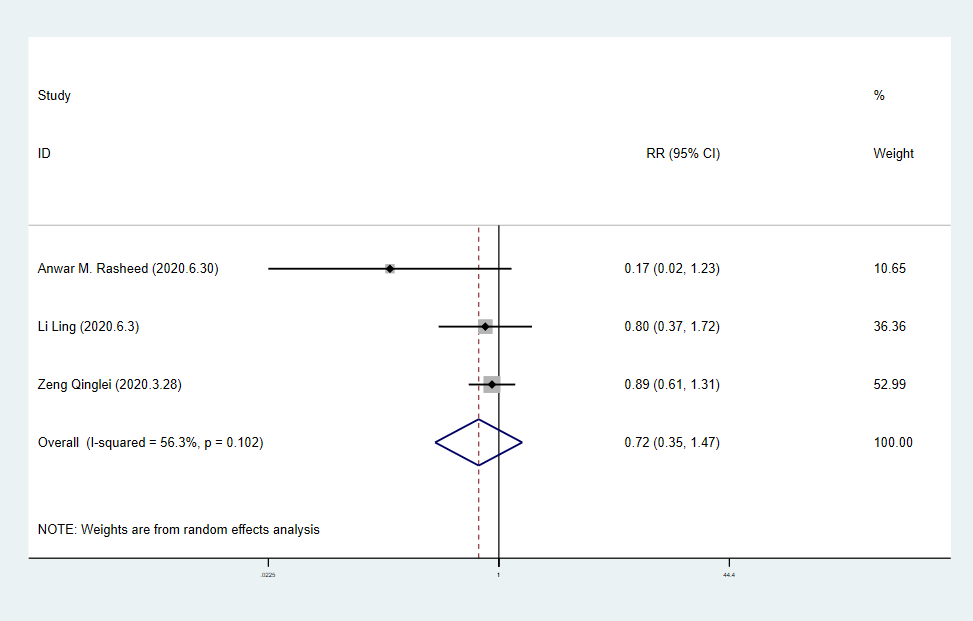
**Forest plot of RR for mortality in critical patients with COVID-19.**

We extracted 10 sets of data from 9 controlled studies to calculate mortality of COVID-19 patients. The results of meta-analysis of mortality are shown in forest-plot (Figure 1) and a fixed-effect model (I^2^=0.0%) was used on the 9 studies. Our study showed that the mortality difference of COVID-19 patients treated with or without CP was statistically significant (RR=0.57 [0.44-0.74]). We assessed publication bias statistically using Egger’s test, showing that publication exists in these 9 articles included (Figure 2). We used trim and fill method with fixed-effects model to further examine the publication bias (Figure 3), and we knew that publication bias did not make an impact on the stability of our study’s results. Considering mortality might alter with severity of the disease although we knew there was no heterogeneity existing in 9 articles (I^2^=0.0%), we performed a subgroup analysis of severe and critical patients. Consistent with our anticipation, the mortality decreased significantly in severely ill patients treated with CP (RR=0.54 [0.36-0.80]) and a fixed-effect model (I^2^=0.0%) was used on 4 studies, as shown in Figure 4. But the mortality of critical patients did not reduce significantly (RR=0.72 [0.35-1.47]) and a random-effect model (I^2^=56.3%) was used on 3 studies, as shown in Figure 5.

### The effect of CP on clinical improvement patients with COVID-19

From the analysis results described previously, we concluded that severe patients were more likely to have higher survival rates. We continued to qualitatively analyze the improvement effect of CP on clinical symptoms in two groups of patients, respectively.

### Patients with severe disease

A randomized clinical trial [34] declared that time to clinical improvement of severely ill patients within 28 days was 4.94 shorter (95%CI, -9.33 to -0.54 days) in the CP group compared with the control group, and that clinical improvement occurred in more patients in the intervention group than in the control group with statistical significance. Similarly with other studies [11, 18, 19, 26], CP therapy did provide an important signal of possible benefit in the severely ill patients. A matched control study stated that the covariates-adjusted odds ratio for worsening oxygenation on day 14 was 0.86 (95%CI: 0.75~0.98; p=0.028), and the CP group also showed a reduction in the proportion of patients with worsened oxygenation status on day 1 and day7 compared with the control group, but it did not have a statistical significance [26].

### Patients with critical disease

Convalescent plasma treatment cannot decrease mortality rate with statistical significance, but can extend survival [8]. In this study, 5 of 6 in CP subgroup died and 14 of 15 in control subgroup died. After CP therapy, the duration of illness, which was calculated from the onset of illness to the date of discharge or death, can be extended significantly (P=0.03). This is similar to the conclusion of the publications that administration of convalescent plasma containing neutralizing antibody was followed by improvement in the patients’ clinical status [9, 13–15, 23], including body temperature, the SOFA score, PaO_2_/FiO_2_, chest X-ray and the laboratory data. In other words, although the clinical endpoint cannot be avoided in patients with COVID-19, the disease course can be improved. To some extent, the prolonged survival time and the improvement of disease course can alleviate the suffering of patients, resembling the role of digitalis in the treatment of cardiac insufficiency.

### The safety of convalescent plasma transfusion

Some significant concerns have been raised concerning the side effects of convalescent plasma in COVID-19, such as transfusion-associated circulatory overload, the infusion of complement proteins and coagulation factors to circulatory system and antibody-dependent enhancement of COVID-19 disease [35]. However, few adverse effects were observed with convalescent plasma transfusion in patients with severe and critical disease, according to publications included in our study [8, 11, 18–21, 23–25, 27, 29, 33, 34, 36]. A study of 5000 hospitalized patients with severe or life-threatening (critical) COVID-19 in the U.S. [16] reported that the incidence of all serious adverse events (SAEs) in the first four hours after transfusion was <1% and the rate of SAEs definitely associated with transfusion was objectively <0.1% of all transfusions. In summary, the paucity of serious adverse effect reduces concerns about potential harm to patients from CP administration.

### The effect of convalescent plasma transfusion on viral load

Studies have reported that viral load was highly related to disease severity and progression [37]. Antiviral drugs inhibit viral reproduction to reduce the harm to health. And virus-specific antibody in the convalescent plasma, which could speed up the clearance of the virus and prevent virus from entering target cells, acts as the primary mechanism for limiting and removing viruses [38]. As reported in articles included in our study, CP-treated patients with severe or critical COVID-19 had large reductions in their serum viral load and a negative conversion of viral PCR occurred in most patients at 72 hours after transfusion [8–15, 17, 18, 25, 33, 34]. Obviously, patients can benefit from plasma transfusion. Viremia generally peaks in the first week after infection in most viral diseases and patients usually develop a primary immune response by day 10-14 followed by virus clearance [39]. Considering the mechanism of antibody, the best time to treat patients with convalescent plasma is in the first week after infection. It also suggests that plasma from recovered patients would be more effective when given early in the course of the disease in clinical management.

## DISCUSSION

According to the literature we included, convalescent plasma transfusion is safe and has unquestionable function in reducing viral load and improving mortality of severely ill patients. Although the decrease in mortality of critically ill patients is not statistically significant, CP does possess therapeutic significance, including improving clinical symptoms. Indications could be obtained from the treatment of cardiac insufficiency, where clinicians devote their efforts to improving the quality of patients’ life by using digitalis. Even if the endpoint of a disease cannot be altered, it is still worthwhile to modify the course.

A review about CP therapy [40] reported that convalescent plasma treatment appeared effective and safe for COVID-19, but there was clearly a need for well-designed RCTs to further evaluate its efficacy and safety. A randomized trial [34] declared only 2 adverse events existed among 52 individuals with treatment of CP. Also, there was a study [16] suggesting that transfusion of convalescent plasma was safe in hospitalized patients with COVID-19. These two studies verified the safety of CP transfusion. The patients were diagnosed with quantitative reverse transcriptase–polymerase chain reaction (qRT-PCR) and viral load was detected again to assess the effect of therapy. We came to a conclusion that viral load decreased and became negative within some days (e.g., 3 days, 12 days or 28 days) after transfusion. Considering the mechanism of antibody and the peak time of viremia, the best time to treat patients with plasma is in the first week after infection. A study [36] reported that no patients died if they were treated with plasma within 7 days of admission, and that the mortality of patients who received transfusion of convalescent plasma after 7 days of hospitalization was 10%, while that of the patients who did not receive plasma was 30%. This suggests that the therapeutic effect of CP transfusion is related to the time of treatment, which is consistent with the mechanism of viral disease. In our analysis stratified by disease severity, among patients with severe disease, the decrease of mortality was statistically significant, and time to clinical improvement was significantly shorter in the treatment group compared with the control group [34]. What’s more, clinical improvement in clinical symptoms and several indicators has been confirmed. In the subgroup of patients with critical disease, the RR of mortality has no statistical significance. Obviously, severe patients benefited more than critical patients. Additionally, the study showed that in a covariates-adjusted Cox model, confirmed association existed between convalescent plasma transfusion and improved survival in non-intubated patients (hazard ratios: 0.19 (95%CI: 0.05 ~0.72); p=0.015), but not in intubated patients [26]. The conclusion of this study also reminded us that CP treatment is more efficient on patients with milder disease. In China, if the current therapeutic strategies are not satisfactory for critically ill patients, physicians might turn to convalescent plasma transfusion based on the Pneumonitis Diagnosis and Treatment Program for SARS-CoV-2 infection (Trial Version 7). And FDA approves use of convalescent plasma to treat critically ill patients [41]. As described earlier, the significance of convalescent plasma administration cannot be ignored and needs to be further studied.

The use of convalescent plasma was associated with clinical improvement without a statistically significant effect on mortality in those patients with critical disease [42], similar with remdesivir. The study [34] reported that CP administration was related to some significant clinical improvement in severe patients but not in critically ill patients with COVID-19. The importance of clinical improvement as a primary endpoint became apparent as the trials progressed for both remdesivir and COVID-19 convalescent plasma [43]. A piece of good news for convalescent plasma administration is that much fewer adverse events occurred in the process of CP use than that of remdesivir [8, 34, 42]. Convalescent plasma treatment and remdesivir, as two potential therapeutic options for COVID-19 have different mechanisms. The former utilizes neutralizing antibodies while the latter is an antiviral drug. It is likely that remdesivir neither one is sufficient enough on its own. The two could thus be synergistic to protect the target cells [44]. A case report stated that the obstetric patient treated with remdesivir and convalescent plasma had had no further issues after discharge [10] and there are some studies suggesting a combination of these two treatments in clinical management [44, 45]. Further trials should consider the effect of combining remdesivir and convalescent plasma on COVID-19.

There are some limitations in our study, despite the significance of our results. Firstly, the available data is limited and the endpoints of different studies are unequal, so we only analyzed mortality of COVID-19 patients roughly. Moreover, as a secondary study, these data (e.g., the method of mortality calculation, drug dosage, laboratory parameters and the specific time of virus turning negative) are not primitive and we could not standardize them. Additionally, clinical studies published about CP use are limited thus far. Hence, the conclusion of this study may be incomplete and further research is needed. We will continue to focus on the progress of the use of convalescent plasma in the future.

In summary, the use of convalescent plasma transfusion is safe and also helps viral loads decrease and become negative for patients with COVID-19. For critically ill patients, CP treatment did not have effects on mortality with statistical significance but could improve the quality of patients’ life to some extent. Severely ill patients benefitted more from the administration of convalescent plasma than critically ill patients. Given that there is no specific drug or vaccine to this disease and the CP administration can benefit patients without serious side effects, we consider that CP has therapeutic potential in COVID-19 so far and further clinical trials are needed.

## MATERIALS AND METHODS

### Search strategy

We searched Pubmed, Web of Science, bioRxiv and medRxiv for studies published newly from December 8, 2019 to August 20, 2020 using the search term “convalescent plasma AND COVID-19”. The study did not require any ethics committee approval. This research was done without patient involvement. Patients were not invited to comment on the study design and were not consulted to develop patient-relevant outcomes or interpret the results. Patients were not invited to contribute to the writing or editing of this document for readability or accuracy.

### Study selection

All literature researched were imported into Endnote X9 software and duplicates were removed. Two reviewers (Luo and Feng) independently screened the studies by title and abstract to exclude those not related to the current study. Publications included met the following criteria: (i) patients were diagnosed with RT-PCR as suggested by WHO; (ii) patients were treated with convalescent plasma and the effectiveness or safety of CP therapy was evaluated; (iii) the type of study was clinical studies including case reports, case series, observational studies, matched-control studies, a proof of concept study and randomized clinical trials.

### Data extraction

We divided all included patients with stated severity into 2 categories that were severely ill patients and critically ill patients, according to the research object. Severe COVID-19 was defined as respiratory distress: ≥30 breaths/min; in resting state, oxygen saturation of 93% or less on room air; or arterial partial pressure of oxygen (PaO2)/fraction of inspired oxygen (FiO2) of 300 or less [34]. Life-threatening COVID-19 was defined as respiratory failure: requiring mechanical ventilation; shock; or other organ failure (apart from lung) requiring intensive care unit (ICU) monitoring [34]. A data extraction protocol was designed by two reviewers (Luo and Feng). The two reviewers extracted the data from the nine eligible clinical controlled studies [8, 34] to analyze the mortality in patients with COVID-19 and differences were resolved by consensus. Additionally, we analyzed the clinical improvement information in all included articles, such as the improvement of blood oxygen saturation and laboratory data, to review the effect of CP therapy comprehensively and we studied all the included literature to discuss the safety of CP therapy by analyzing the adverse effect qualitatively.

### Statistical analysis

Statistical analyses were performed with STATA (version 15.1; Stata Corporation, College Station, TX, USA) for studying the mortality of patients with COVID-19. The pooled frequency with calculation of relative ratio (RR) and 95% confidence interval (CI) of RR were assessed using a fixed-effect model. The between-study heterogeneity was assessed by the I^2^ statistic. Publication bias was assessed statistically by using Egger's tests (p<0.05 was considered indicative of statistically significant publication bias). Subgroup analysis was also performed.

### Quality assessment

We assessed the quality of the RCT and clinical controlled studies included for analyzing the mortality by Cochrane collaboration’s tool for assessing risk of bias [46] and Newcastle-Ottawa Scale (NOS) [47], respectively.

### Dissemination declaration

Dissemination to results to the study participants and or patient organizations is not possible/applicable.
